# Instability in progressive multiple sequence alignment algorithms

**DOI:** 10.1186/s13015-015-0057-1

**Published:** 2015-10-09

**Authors:** Kieran Boyce, Fabian Sievers, Desmond G. Higgins

**Affiliations:** Conway Institute of Biomolecular and Biomedical Research and UCD School of Medicine and Medical Science, University College Dublin, Dublin 4, Ireland

**Keywords:** Clustal, Kalign, Mafft, Muscle, Pfam, Multiple sequence alignment, Large scale alignment, Sequence order

## Abstract

**Background:**

Progressive alignment is the standard approach used to align large numbers of sequences. As with all heuristics, this involves a tradeoff between alignment accuracy and computation time.

**Results:**

We examine this tradeoff and find that, because of a loss of information in the early steps of the approach, the alignments generated by the most common multiple sequence alignment programs are inherently unstable, and simply reversing the order of the sequences in the input file will cause a different alignment to be generated. Although this effect is more obvious with larger numbers of sequences, it can also be seen with data sets in the order of one hundred sequences. We also outline the means to determine the number of sequences in a data set beyond which the probability of instability will become more pronounced.

**Conclusions:**

This has major ramifications for both the designers of large-scale multiple sequence alignment algorithms, and for the users of these alignments.

## Background

The creation of a multiple sequence alignment is a routine step in the analysis of homologous genes or proteins. For aligning more than a few hundred sequences, most methods use a heuristic approach termed “progressive alignment” by Feng and Doolittle [1]. This is a two-stage process: first a guide tree [2] is created by clustering the sequences based on some distance or similarity measure, and then the branching structure of the guide tree is used to order the pairwise alignment of sequences. The power of progressive multiple sequence alignement may come from the fact that “more similar” sequences are aligned first: “...assuming that in progressive alignment, the best accuracy is obtained at each node by aligning the two profiles that have fewest differences, even if they are not evolutionary neighbours” [3].

The guide tree determines the order in which the sequences are aligned. All sequences are compared to each other to generate a matrix of distance measures between each pair of sequences. By necessity, the calculation of these distance measures must be fast as it will clearly require $\mathcal {O}(N^2)$ time and memory for *N* sequences. Most alignment programs use *k*-tuple scores [4, 5] to measure the similarity of two sequences, or related word-based measures. Some use other string-matching algorithms to the same effect. While these approaches are fast, they only score exact matches between two sequences. For proteins, amino acids that are considered very similar, for example using the PAM [6] or BLOSUM [7] matrices, are treated as complete mismatches.

This paper examines the impact of the tradeoff of accuracy for speed in the construction of the guide trees in protein progressive multiple sequence alignment. We find that, because of a loss of information when calculating the distance measures, the alignments generated are inherently unstable. This instability is easily seen by changing the order of the protein sequences in the input file. This will cause a different alignment to be generated. We also show that, while this instability is more apparent with larger alignments and with some alignment programs, it is also found in small alignments of less than 100 sequences.

This instability is due to huge numbers of tied scores in the distance matrices used to make the guide trees. With word-based distances, there is a relatively small number of possible distance scores that can be found between two sequences. This number will depend on the length of the sequences and on the metric used. The effect is that once you get to even moderately large numbers of sequences, the distance matrix will have many tied scores which would ideally be represented in the guide tree as multifurcations. Progressive alignment is a strictly pairwise algorithm and the branching order within these tied groups will be completely arbitrary and determined purely by how the clustering code was written. If you change the sequence order, you will change the cluster order and hence the order of progressive alignment. This means that the supposed power of the guide tree to sensibly align the sequences in the correct order is lost and the considerable computation effort required to calculate them may be completely wasted.

## Methods

### HomFam

The analysis presented here uses the HomFam alignment benchmark system [8]. This consists of the single-domain Pfam [9] (version 25) families which have at least 5 members with known structures in a HOMSTRAD [10] structural alignment. We measure the proportion of correctly aligned core columns out of all aligned core columns in the reference sequences (BAliSCORE TC score [11]), when these sequences are embedded in larger data sets. The TC score ranges from 0.0 (no core columns in the reference sequences correctly aligned) to 1.0 (all reference sequence core columns correctly aligned). An alternative TC score measures the proportion of all correctly-aligned columns. While the results were similar, we use core columns in this paper.

On examining the HomFam sequences, it was noticed that a number of proteins had the same amino acid sequence even though they were (correctly) labelled differently in Pfam. As an example, in the zinc finger family (Pfam accession number PF00096), the sequence information for:

 >D2I3U5_AILME/95-116

ACADCGKTFSQSSHLVQHRRIH

and

>ZN787_HUMAN/95-116

ACADCGKTFSQSSHLVQHRRIH

are identical. Table 1 shows the number of sequences in each HomFam family and the number of these which are unique. In the remaining analysis, duplicate sequences were removed from the HomFam families. Having duplicate sequences will automatically give tied distances and we wished to separate this effect from effects due to using *k*-tuple scores.

**Table 1:** Duplicate sequence percentages in HomFam protein families

Protein family	Total seqs	Unique seqs	% Dup
aadh	3119	2348	24.72
aat	25,090	19,879	20.77
Acetyltransf	46,279	31,943	30.98
ace	3983	3787	4.92
adh	21,326	15,452	27.54
aldosered	13,270	10,787	18.71
Ald_Xan_dh_2	2583	2037	21.14
annexin	3133	2288	26.97
asp	3249	2979	8.31
az	1057	892	15.61
biotin_lipoyl	11,826	7332	38.00
blmb	17,194	13,102	23.80
blm	9097	7145	21.46
bowman	494	218	55.87
cah	1374	1197	12.88
ChtBD	769	447	41.87
cryst	1153	909	21.16
cyclo	6282	4967	20.93
cys	4303	3910	9.13
cyt3	379	347	8.44
cytb	3200	2622	18.06
DEATH	1176	874	25.68
DMRL_synthase	2094	1423	32.04
egf	7762	5405	30.36
flav	4606	3103	32.63
GEL	2190	1583	27.72
ghf10	1497	1393	6.95
ghf11	516	461	10.66
ghf13	12,597	9870	21.65
ghf1	4350	3471	20.21
ghf22	748	608	18.72
ghf5	2711	2355	13.13
glob	3942	2828	28.26
gluts	10,085	7841	22.25
gpdh	7683	4993	35.01
hip	162	115	29.01
hla	13,460	9148	32.03
HLH	6776	3417	49.57
HMG_box	4774	2988	37.41
hom	12,029	6044	49.75
hormone_rec	3504	2896	17.35
hpr	3344	1878	43.84
hr	3702	1985	46.38
icd	5673	4505	20.59
il8	1062	799	24.76
ins	787	524	33.42
int	7567	6185	18.26
KAS	2064	1490	27.81
kringle	1082	821	24.12
kunitz	2256	1753	22.30
ldh	7353	3094	57.92
LIM	6423	3729	41.94
ltn	1056	909	13.92
lyase_1	7627	5611	26.43
mmp	1421	1136	20.06
mofe	2561	2326	9.18
msb	4876	4094	16.04
myb_DNA-binding	10,393	7124	31.45
OTCace	4790	3234	32.48
oxidored_q6	3343	1974	40.95
p450	21,001	19,700	6.19
PDZ	14,944	9552	36.08
peroxidase	4509	3589	20.40
phc	2945	1961	33.41
phoslip	928	803	13.47
profilin	682	579	15.10
proteasome	5715	4549	20.40
Rhodanese	14,043	10,011	28.71
rhv	17,970	9151	49.08
ricin	740	548	25.94
rnasemam	492	438	10.98
rrm	27,590	18,692	32.25
rub	1430	975	31.82
rvp	93,675	64,987	30.62
scorptoxin	355	311	12.39
sdr	50,144	40,212	19.81
seatoxin	88	63	28.41
serpin	3136	2957	5.71
slectin	927	749	19.20
sodcu	2031	1586	21.91
sodfe	4447	2728	38.65
Stap_Strp_toxin	634	174	72.56
sti	608	536	11.84
subt	7506	6469	13.81
Sulfotransfer	2484	2269	8.65
tgfb	1598	1022	36.04
tim	3894	2909	25.30
tms	2113	1518	28.16
TNF	551	417	24.32
toxin	488	450	7.79
trfl	830	742	10.60
tRNA-synt_2b	11,288	7670	32.05
uce	4545	3744	17.62
zf-CCHH	88,330	45,901	48.03

The list of HomFam protein families, the total number of sequences in each family, the number of unique sequences, and the percentage of the total number of sequences that are duplicates

One side effect of the removal process is that the remaining sequences are sorted in ascending alphabethical order of the sequences (not the sequence names) within each of the HomFam families. As each dataset is later randomly shuffled before being aligned, this will not have an effect on any of the alignments produced.

### Software

This article examines the instability of the alignments produced by the progressive multiple sequence alignment programs Clustal Omega [12], Kalign [13], Mafft [14] and Muscle [3]. These programs were selected based on their widespread use, their ability to align more than a thousand protein sequences, and their use of a guide tree based on the similarity between each pair of sequences to determine the order in which the sequences will be aligned.

Each of the alignment programs generates a distance matrix containing the similarity or distance measures between all pairwise combinations of input sequences. Kalign does not output this distance matrix by default, but on examining kalign2_main.c line 135, the code to output the distance matrix has been commented out. This code was uncommented and modified to output the distance matrix to a specific text file. In addition, the distance measures were output to 25 decimal places to ensure that any duplicates were not as a result of rounding when formatting the output.

The other three alignment programs were also modified to output distance measures to 25 decimal places: Clustal Omega: line 327 of clustal/symmatrix.c; Mafft: line 2643 of io.c; Muscle: line 59 of fastclust.cpp.

For all four alignment programs, the runtime parameters were limited to those required to generate a distance matrix. By default, Clustal Omega uses the mBed algorithm [8] to cluster the sequences on the basis of a small number of “seed” sequences. This only requires the calculation of the similarity measures between these seed sequences and all other sequences in the input file. By requesting that a full distance matrix be generated and output, the sequences were clustered using the similarity measures between all pairs of input sequences.

**Fig. 1:**
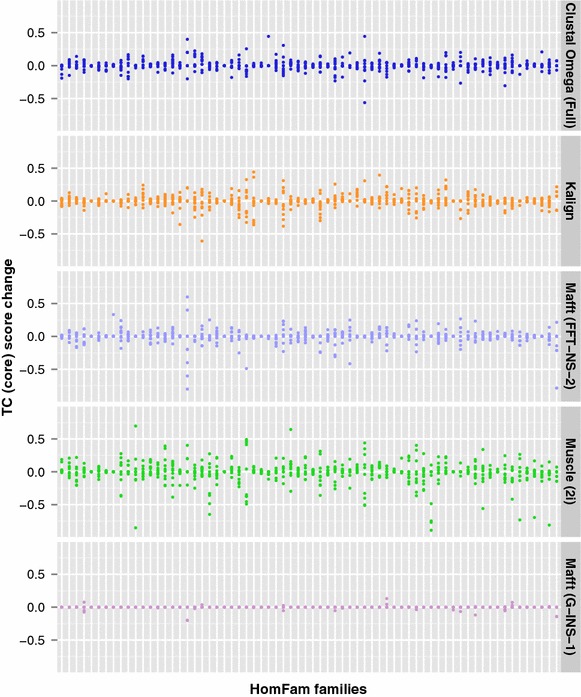
Difference in TC core scores for random samples and in reverse order. The difference in the TC core scores for 1000 randomly-selected sequences and in reverse order. 68 HomFam protein families. $n=10$ samples per family

For Mafft, the FFT-NS-1, FFT-NS-2 and G-INS-1 algorithms were used. With FFT-NS-1, a distance matrix is first generated using the 6-tuple score between each pair of sequences—both sequences are scanned from the start for matching 6-tuples, and when a match is found the score is incremented and scanning continues from the next residue [4]. A guide tree is then constructed by clustering according to these distances, and the sequences are then aligned using the branching order of the guide tree. With FFT-NS-2, the alignment produced by the FFT-NS-1 method is used to regenerate the distance matrix and the guide tree, and then do a second progressive alignment. In this paper, FFT-NS-1 will be specified whenever distance measures are needed. If no distance measures are required, the default FFT-NS-2 method will be used. The G-INS-1 algorithm was also used in Figure1 for comparison with a distance measure that doesn’t rely on matching *k*-tuples.

With Muscle, the number of iterations was limited to 2 rather than the default of 16. This is the number of iterations recommended by the authors for large datasets.

The program versions and runtime parameters used are as follows:

Clustal Omega (v1.2.0-r289): --full --distmat-out=...

Kalign (v2.04): -q

Mafft (v7.029b) FFT-NS-1: --retree 1 --anysymbol --distout

Mafft (v7.029b) FFT-NS-2: --anysymbol --distout

Mafft (v7.029b) G-INS-1: --anysymbol --globalpair

Muscle (v3.8.31): -maxiters 2 -DistMx1 ...

### Supporting material

A package of utility programs, data files and scripts is available for download from http://www.bioinf.ucd.ie/download/2015instability.tar.gz.

## Results and discussion

In the following sections, we refer to distance matrices and the calculation of distances between sequences. In most of the cases we discuss here, we actually use similarity scores. Nonetheless these can be easily converted to distances and we retain the use of the words distance and distances out of convenience.

### Alignment instability

For each of the 94 HomFam families we selected the HOMSTRAD reference sequences and a random selection of sequences to make up 1000 sequences in total. Families with an insufficient number of sequences were excluded, leaving a total of 68 families.

The 1000 sequences were randomly shuffled, a default alignment was generated (for Mafft, both the FFT-NS-2 and G-INS-1 algorithms were used), and the alignment quality measured using its BAliSCORE TC score. The order of the sequences in the input file was then reversed, the alignment repeated with the same parameters and the quality of this alignment measured. The difference between the two quality scores was then calculated. This process was repeated 10 times for each of the 68 HomFam families, and the results are presented in Fig. 1.

In the first four panels, and for virtually all of the represented HomFam families, reversing the order in which the sequences are listed in the input file has an impact on the quality of the alignments produced. For some protein families and alignment programs this impact is considerable, with the alignment of up to 50 % of columns in the reference sequences changing by reversing the order of the input sequences. In the fifth panel, Mafft G-INS-1 uses Needleman-Wunsch [15] to calculate the distance measures between each pair of sequences. Although some instability is still present, it is significantly lower than for the other alignment programs using their default parameters.

It should be noted that Mafft’s G-INS-1 is considerably slower than FFT-NS-2 for the given number of sequences, taking approximately two orders of magnitude longer to run. It also requires over ten times more memory, and both memory and time requirements scale quadradically. As a result it is not recommended for aligning more than a few hundred sequences, but was included in the figure for reference purposes. In the remainder of this paper, we will only examine the distance measure calculations used when aligning larger numbers of sequences.

### Unique distances

Clustal Omega uses 1-tuple scores to determine the distance measures between proteins, where the scores are calculated in the same was as Mafft’s 6-tuple score except for the different lengths of matching string. Muscle uses 6-tuple scores calculated in the same way as Mafft, and Kalign uses the Muth Manber [16] approximate string matching algorithm. Such methods essentially count the number of matches between sequences, ignoring both the position of the matches and the actual values matched. The number of matches between sequences is therefore related to the lengths of the sequences. Clearly different, unrelated pairs of sequences can generate the same distance. In addition, the chances of seeing such matches will increase as the number of sequences being aligned increases. It is not clear how the clustering algorithm used in each of the alignment programs resolves such ties in distance measures. However, unless this scenario is specifically catered for, the default approach will be to choose between pairs of sequences based on their positions in the input file, either the first pair with that distance measure or the last pair.

**Fig. 2:**
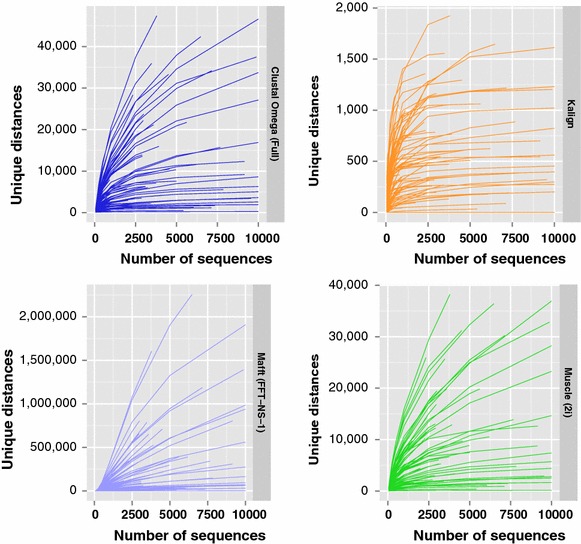
Unique distances by number of sequences for each alignment program. The number of unique distances with increasing number of sequences. Each *line* is the mean of 100 samples for each HomFam protein family

In order to further investigate the frequency of such tied distances, the number of unique distance values in a distance matrix computed for a dataset was determined. The four alignment programs, Clustal Omega, Kalign, Mafft and Muscle were run with the parameters listed previously on random samples of sequences drawn from each of the HomFam protein families. Sample sizes ranged from 50 to 10,000 sequences (or as many unique sequences as were in the HomFam family), and each sampling was repeated 100 times. The number of unique distances were counted in the distance matrices produced from each alignment, and the mean number of unique distances for each family and number of sequences are presented in Fig. 2.

The number of unique distances generated by Mafft is considerably higher than for the other alignment programs. However, for all alignment programs, the numbers of unique distances show clear trends of levelling off as the number of sequences increases. In addition, as the total number of distances calculated is given by $N(N-1)/2$ for *N* sequences, for the larger data sets the vast majority of distance measures are duplicated in each alignment program.

### Same length sequences

To determine why the number of unique distances reaches a plateau while the total number of pairwise distances increases quadratically, we need to examine how the distances between sequences are calculated. To simplify the analysis, we will first look at sequences of the same length.

Clustal Omega uses 1-tuple scores for comparing sequences. With sequences of the same length it can therefore only generate a maximum of $L+1$ unique distances where *L* is the length of the sequences. These correspond to sequences with no matches, 1 match, 2 matches, etc. up to identical sequences. Mafft and Muscle use 6-tuple scores, so the maximum number of unique distances between sequences of length *L* is $(L-5)+1$ where $(L-5)$ is the number of 6-tuples in a sequence and the additional $+1$ is necessary if no matches are found. The calculation of these distance measures ignores both the position of the matches and the values matched.

Depending on the actual amino acids, Kalign calculates the distance measure as zero between pairs of protein sequences of up to 32 amino acids each.

### Different length sequences

Clustal Omega scales the 1-tuple scores by the length of the shorter of the two sequences. Similarly Muscle scales its 6-tuple scores by the number of 6-tuples in the shorter sequence, and Kalign scales based on the length of the longer sequence. In Mafft, the distance measure is calculated as:

$$\begin{aligned} D'_{ij} = D_{ij}/f(x,y) \end{aligned}$$

where:

$$\begin{aligned} D_{ij} = 1 - \frac{S_{ij}}{min(S_{ii},S_{jj})} \end{aligned}$$

$$\begin{aligned} f(x,y) = \frac{y}{x} \times 0.1 + \frac{10000}{(x + 10000)} + 0.01 \end{aligned}$$

$S_{ij}$ is the 6-tuple score between sequences *i* and *j*, and *x* and *y* are the lengths of the longer and shorter sequences respectively. The additional scaling is deemed necessary as $D_{ij}$ can be near zero when comparing very short and very long sequences, even if the sequences are unrelated.

### Theoretical maximum number of unique distances and sequences

Based on this analysis, the two factors that determine the number of different possible distance measures are the lengths of the sequences and the number of different sequence lengths. For simplicity, we will ignore the minor adjustments to the sequence lengths due to using 1-tuples or 6-tuples. Hence, for Clustal Omega, Kalign and Muscle, the theoretical maximum number of unique distances is given as the product of the longest sequence length and the number of different sequence lengths in the dataset. For Mafft, as both sequence lengths are included in the additional scaling factor, the theoretical maximum is the longest sequence length times the square of the number of different sequence lengths. These theoretical maxima are conservative as all sequences may not be as long as the longest sequence, and all possible matches for all sequence lengths may not be found.

So, for Clustal Omega, Kalign and Muscle:

$$\begin{aligned} MaxUniqueDists = MaxSeqLength \times Count(SeqLengths) \end{aligned}$$

and for Mafft:

$$\begin{aligned} MaxUniqueDists = MaxSeqLength \times Count(SeqLengths)^2 \end{aligned}$$

where *MaxUniqueDists* is the theoretical maximum number of unique distances, *MaxSeqLength* is the length of the longest sequence in the dataset, and *Count*(*SeqLengths*) is the number of different sequence lengths.

In addition

$$\begin{aligned} MaxSeqs(MaxSeqs - 1)/2 = MaxUniqueDists \end{aligned}$$

where *MaxSeqs* is the maximum number of sequences that can be aligned before duplicate distance measures are generated.

**Fig. 3:**
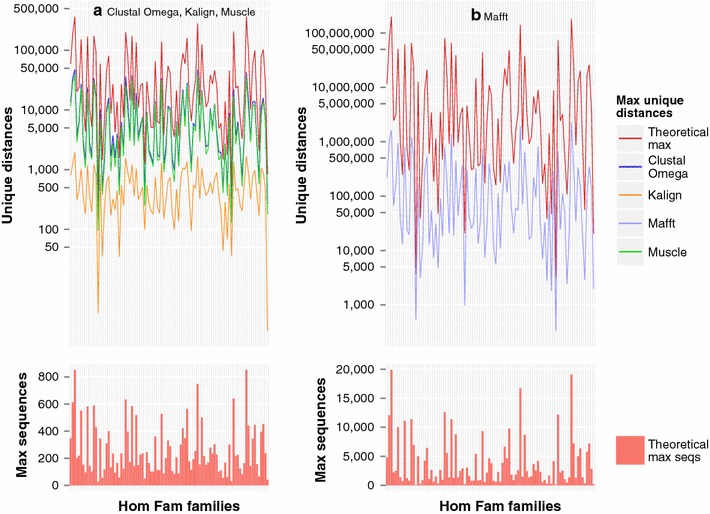
Theoretical maximum and actual number of unique distances, and maximum theoretical number of sequences that can be aligned without duplicate distance measures. The theoretical maximum number of unique distances for each HomFam family, the actual number of unique distances found in the datasets used to generate Fig. 1, and the maximum theoretical number of sequences that can be aligned without generating duplicate distance measures based on the calculation that *N* sequences will produce $N(N-1)/2$ distance measures

Figure 3 plots these theoretical maxima for Clustal Omega, Kalign and Muscle (3a), and Mafft (3b) for each HomFam family based on all sequences in each family. Also shown are the maximum numbers of unique distances for each family found in the datasets used to construct Fig. 2 previously. As can be seen, the pattern of unique distances in the datasets follows but is lower than the theoretical maxima.

The lower plots in Fig. 3 shows the maximum number of sequences that can be aligned without duplicate distance measures, derived from these maximum numbers of unique distances. Again these maximum numbers of sequences are a conservative measure, as they are based on all lengths of sequences occurring in the dataset and each sequence having its full range of possible matches. Perhaps the most stiking thing about the lower plot is that the numbers of sequences are so low, particularly for Clustal Omega, Kalign and Muscle.

It should be noted, however, that duplicate distance measures do not necessarily lead to instability in the alignment generated. It will depend on whether the duplicate measures are the lowest values in the distance matrix at that step in the clustering process, which will in turn depend on what has happened in the previous clustering steps. Hence, we cannot say for definite that duplicate measures will lead to alignment instability. However, as the number of duplicate measures increases, so too does the likelihood of alignment instability. As the alignment instability is determined by the characteristics of the input sequences, we recommend that alignment programs be modified to issue a warning of potential instability when the clustering algorithm encounters tied distance measures.

### Smaller alignments

**Fig. 4:**
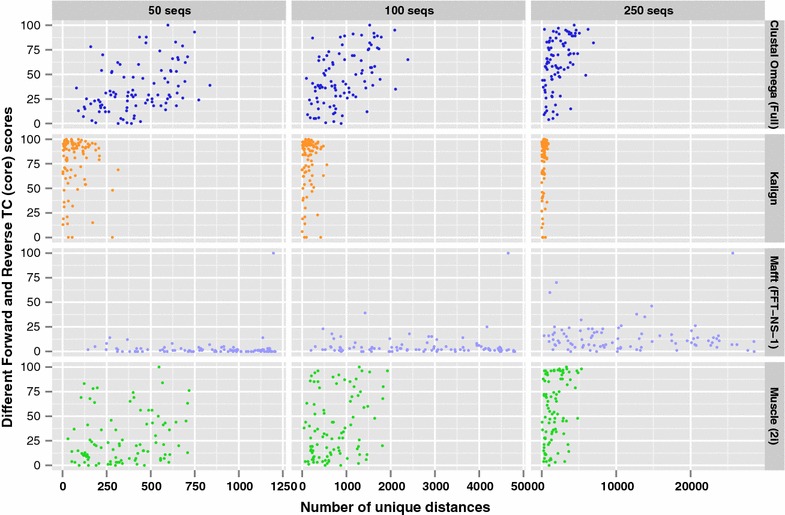
Count of the differences in forward and reverse TC core scores. The number of samples within each HomFam family where the forward and reverse TC core scores are different. $n=100$ samples for each family and dataset size

While the instability demonstrated earlier is more apparent in larger alignments, it can also be present when smaller numbers of sequences are aligned. This can be shown by randomly selecting 50, 100 and 250 sequences (including each family’s reference sequences) from each HomFam family and calculating the TC scores for the forward and reversed datasets, as was done in Fig. 1. 100 random samples were used for each HomFam family and for each of the three dataset sizes. For each sample, the forward and reverse TC scores were compared, and the number of differences for each HomFam family were counted. These counts are shown in Fig. 4.

As can be seen, the instability in sequence alignments occurs even with small alignments. Also, as the number of sequences increases so too does the number of differences in TC scores. While there is no clear trend between the number of unique distances and the number of TC score differences for a particular alignment program, this trend can be seen across the different programs—Mafft shows the fewest number of differences in TC scores and Kalign the most.

### Algorithm symmetry

It should also be pointed out that another reason for the difference in TC scores reported above may be due to the asymmetry of the different implementations of distance measure calculations. Different distance measures could then cause a different clustering order and give a different tree topology, causing sequences to be aligned in a different order.

To illustrate, we randomly select two Retroviral aspartyl protease (Pfam accession number PF00077) sequences, run the four alignment programs and extract the distance measures between the two sequences. The order of the two sequences is then reveresed, the alignment programs run again, and the distance measure from this second run is compared with the original. (Clustal Omega requires a minimum of three sequences, so three sequences were selected at random and the distances between the first and third sequences were compared.)

Out of 10,000 samples, for Clustal Omega there were 9 different distances identified. With Mafft and Muscle, no different distance measures were found. However, with Kalign 6516 differences were found.

## Conclusions

In this paper we have demonstrated a very strong dependence on the order of the input sequences in a data file when we measure multiple alignment accuracy. This effect is disconcerting as merely changing the order of the sequences can change the alignment. The scale of this effect is somewhat surprising and mainly shows up when the numbers of sequences grows large. It can, nonetheless, be seen in data sets of the order of a hundred sequences or so.

We have also noticed that when we examine distance matrices generated by some widely used MSA packages that these become increasing dominated by tied values. The more sequences you have, the greater the percentage of the scores in a distance matrix that are duplicates of other scores. We can trace this effect to the use of *k*-tuple scores for computing these distances. For sequences of a given length, there is a finite and relatively small number of possible scores that can be generated. For shorter length sequences, the number of possible distances is also reduced. If you use real alignment scores using an amino acid weight matrix such as BLOSUM [7], the number of possible scores is still finite although much greater than with *k*-tuple distances. Given enough sequences though, you will inevitably get many tied values in a distance matrix. The use of such alignment scores is limited however, to relatively small datasets as they are expensive to compute, as was seen with Mafft G-INS-1 in Fig. 1. For really big alignments, of many thousands of sequences, we have little alternative to the use of *k*-tuple or word based scores at some stage of the progressive alignment procedure. Iteration, as carried out by Clustal Omega, Mafft and Muscle can help as the later alignments can use real alignment scores but these are very expensive computationally and do not eliminate tied scores. It is also possible to mitigate the alignment instability by, say, ordering the input sequences lexicographically before calculating the *k*-tuple scores. However, while this will result in a consistent alignment being produced, it is difficult to justify from a biological point of view why one particular alignment out of numerous alternatives should be chosen. The solution to this issue is not clear-cut. We have previously shown [17] that the accuracy of progressive alignment decreases markedly with very large datasets. We assumed this was due to the greedy nature of the algorithm. Here we show that progressive alignment also produces alignments that have a strong dependence on the sequence order in the input file. The use of “chained” guide trees [18] can help improve accuracy but will still have a strong dependence on input file sequence order.
